# Deprescription in Advanced Cancer Patients

**DOI:** 10.3390/pharmacy6030088

**Published:** 2018-08-21

**Authors:** Ferraz Gonçalves

**Affiliations:** Palliative Care Service, Instituto Português de Oncologia, R. Dr. António Bernardino de Almeida, 4200-072 Porto, Portugal; ferrazg@ipoporto.min-saude.pt

**Keywords:** deprescription, polypharmacy, inappropriate medication

## Abstract

The use of multiple drugs is often referred to as polypharmacy, although this term has not been precisely defined. Frequently, drugs are used unwisely in multiple combinations increasing the risk of adverse reactions, or for the long-term prevention of diseases in patients with a short life expectancy who, therefore, will not benefit from them. The reflection on this has led to the introduction of the concept of deprescription. There are many reasons for the inappropriate drug prescription and barriers to reduce medications. Tools were developed to help prescribers to limit the number of prescribed drugs that patients are taking. Several studies have shown that deprescription of some drugs is possible and safe and can even have a positive influence on wellbeing, cognitive function, falls, and admission to a hospital. Deprescription should be individualized and carried out, as far as possible, in agreement with patients and their families. A six-step method for deprescription is proposed.

## 1. Introduction

The use of multiple drugs is often referred to as polypharmacy, although this term has not been precisely defined. Polypharmacy can be defined by the number of drugs, usually five or more, or by the appropriateness of the prescription [1]. The definitions are not limited to prescribed drugs, as many individuals take over-the-counter medicines. The origins of patients receiving polypharmacy are diverse, such as multiple comorbidities, guidelines for the treatment of diseases that may not be applicable in patients with a short life expectancy, unrealistic expectation of patients and carers or the misinterpretation of side effects of the medication as problems requiring more drugs. Patients may also take complementary/alternative therapies. Therefore, often patients are taking multiple drugs, sometimes without any rational purpose for some of them and with potentially dangerous consequences such as adverse effects, cognitive impairment, falls, hospitalization, and even death. It was observed in an emergency department that the risk of adverse drug reactions increased with the number of prescribed drugs, from 13% for two to 82% with seven or more [2]. Multiple drugs may also influence patients’ adherence to treatment and contribute to increased health costs.

Scott et al. defined deprescription as “a systematic process of identifying and discontinuing drugs in instances in which existing or potential harms outweigh existing or potential benefits within the context of an individual patients’ goal, current level of functioning, life expectancy, values, and preferences” [3]. It should be noted that the appropriateness of drug prescription is relative. Drugs may be appropriate in a certain phase of a disease or at a certain age, but not in others; for example, at an advanced age or in patients with a short life expectancy, such as advanced stage cancer. Drugs with a long “time until benefit”, sometimes needing years before their benefit is achieved [4], prescribed with the intention of preventing long-term consequences of conditions such as hypertension, diabetes, dyslipidemia, or others, may make no sense when there is no longer any long-term perspective. In these patients, drugs which serve exclusively to prevent future events should not be used, and only drugs with a symptomatic benefit should be prescribed.

Patients with cancer may live many years and therefore the prevention of future consequences of diseases, such as hypertension, diabetes mellitus, and others may be appropriate. Therefore, it is very important to estimate the life expectancy to select patients who would benefit from deprescription of preventive medication. It should also be noted that cancer patients, particularly the older ones, can have many comorbidities, therefore, can be taking medications for other diseases that may be, or not, appropriate for the stage of the oncological disease. It is also important to differentiate the drugs for comorbidities that have a symptomatic role, such as some drugs for heart failure, and preventive drugs which may be inappropriate depending on the stage of cancer.

## 2. The Magnitude of the Problem

This article focuses on deprescribing in advanced cancer patients. However, many of those patients have comorbidities and are taking medication to prevent the consequences of those diseases.

A study carried out on terminal cancer patients found that potentially futile medications were being taken by patients [5]: gastric protectors (51%), antihypertensive (47%), and antidiabetic agents (11%), according to the Fede et al. criteria [6]. In a study carried out by us in a palliative care service [7], in 448 patients with a median survival of 15 days (range: 1–404), using the Fede et al. criteria when applicable at admission, we found that statins met futility criteria in 97% of cases, gastric protectors in 50%, antihypertensive agents in 27%, antidiabetic prescriptions in 1%, bisphosphonates in 26%, and antidementia drugs in 100% of patients.

Lipid-lowering drugs, especially statins, are the most commonly used preventive medication as several studies have shown [8,9], and the probability of their discontinuation does not seem to be influenced by the diagnosis of a life-threatening disease. For example, data show that statins are prescribed in 62% of cancer patients with a poor prognosis and in 31% of cancer patients within 30 days of death [7].

## 3. Barriers to Deprescription

Prescribing and deprescribing are two sides of the same coin. The pharmacological treatment of a patient should be a dynamic process that should be adapted to their evolving condition, adding and suspending drugs in face of the needs, but the suspension of chronic medication seems to be particularly difficult. Although, other professionals may influence prescription and deprescription, as I mention below, doctors have a key role to play.

### 3.1. Doctors’ Attitudes

A recent systematic review of doctors’ attitudes allows the problems involved in this issue to be synthesized [10]. One is the doctors’ awareness of the appropriateness of drug prescriptions, mainly for individual patients, even when doctors know that the prescription should generally be avoided. Inertia is also a cause for maintaining the prescription of inappropriate drugs and may arise from the fear of negative or unknown consequences for the doctor, such as threatened therapeutic relationship, litigation, or conflict with other health professionals, including other prescribers, consequences for patients, such as withdrawal syndrome, or the occurrence of a negative consequence for which the medication had been prescribed [10]. There is also the belief that drugs have no significant adverse effects. Sometimes the doctor does not want to withdraw the medication prescribed by a colleague who had been treating the patient, particularly if it were prescribed by a specialist. The difficulty in balancing the benefits and harms of drugs, especially when it comes to older people with multiple morbidities, may also contribute to inappropriate prescriptions and polypharmacy.

### 3.2. Other Professionals

There are other professionals, such as nurses and pharmacists, who may influence deprescription, positively or negatively as their requests may also contribute to inappropriate prescriptions [10]. Nurses’ prescribing permission differs among countries and in some of them they have no permission at all. Even so, they may influence prescribing because they observe and can communicate to physicians the treatment burden associated with polypharmacy, particularly those with elevated levels of frailty [11]. For example, the observation of drugs side effects, swallowing difficulties, the number of tablets taken together, the expenses with drugs, monitoring requirements, and refusals to take a medicine and why [11]. These reasons are essential clinical information to be communicated to physicians or discussed in team meetings and may contribute to the suspension of some drugs. Nurses may also have an important role providing advice to patients about various aspects of their prescribed medication and even in over-the-counter medications [12].

Pharmacists also have a role in deprescribing. Their role may be different in different countries, but they are often an underutilized resource in monitoring patient welfare. Pharmacists can suggest the suspension of drugs that are not indicated or contra-indicated for the patient’s condition. One study showed that doctors accept a high percentage of the suggestion from pharmacists [13].

### 3.3. Patients’ Views

A recent systematic review on patients’ views on deprescription [14] showed that patients feel an improvement when they start taking the drug or hope for a future improvement and for that reason they do not want to discontinue the drug. Some patients think that they are doing something that can help their condition and in doing so they feel reassured. Communication problems with their physicians on why and how to cease a medication and lack of support following cessation are also issues identified by patients concerning the withdrawal of drugs. These are allied to pressure from family and/or health professionals and the fear of cessation related to concerns such as return of the previous condition and withdrawal reactions [14].

## 4. Tools for Determining Inappropriate Medication

As deprescription can involve some difficulties as described above, several tools have been developed to assist physicians. Most of those tools were developed for older people and not specifically for cancer patients and one of them, the American Geriatrics Society Beers Criteria for Potentially Inappropriate Medication Use in Older Adults, developed for use in all patients aged 65 or older in the United States, even excludes hospice and palliative care patients [15]. These tools, while not specific to cancer patients, may help in deprescription in this population.

Fede et al. [6] created criteria specifically for advanced cancer patients for the withdrawal of statins, gastric protectors, antihypertensive agents, antidiabetic agents, and, in a less defined way, for any other medication (no clear medical indication identified). However, the most defined criteria are very conservative. For example, the criterion for cessation of antihypertensive agents was “when the patient registered arterial blood pressure <90/60 mmHg at the time of last consultation and symptoms of hypotension”, but probably many more patients with higher arterial blood pressure will not benefit from the medication when survival is relatively short due to advanced cancer. The same could be said about antidiabetic medication. A different tool, the OncPal deprescribing guideline was developed based on the current literature for the de-escalation of medications by systematically reviewing each medication class according to the EphMRA (European Pharmaceutical Market Research Association) anatomical classification list [16]. This tool showed 94% concordance with a panel of experts. The reliability of these tools has not been adequately studied.

## 5. Deprescribing in Advanced Cancer Patients

Deprescribing in advanced cancer patients has much in common with other advanced chronic diseases and old and frail patients. Deprescription of some drugs can be successfully achieved as shown by several studies in different settings. For example, one study including adults with a life expectancy between one month and one year, randomly divided between whether to continue or discontinue statins, suggested that stopping statins is safe and may be associated with benefits including quality of life, use of fewer non-statin medications and a corresponding reduction in medication costs [17]. The influence of drug reduction manifests itself through reduced referral to acute care facilities and one-year mortality or by halving the risk of falls [18]. There may also be an improvement in cognition and daily function with the discontinuation of psychotropic drugs. It was also found that most patients taking antihypertensive drugs remained normotensive after the withdrawal of the specific medication [18].

The most adverse effects resulting from deprescription are the exacerbation of the underlying condition, most frequently associated with cardiovascular drugs, prescribed for hypertension, angina, and heart failure, followed by central nervous system drugs, especially benzodiazepines [18]. The number of drugs discontinued increases the likelihood of adverse effects [18] but, in one study, it was possible to discontinue 58% of medications; only 2% were restarted because of the occurrence of the original indication and 88% of patients reported a global improvement in health [19].

Cancer patients face an additional question which is: when to stop anticancer therapy? Sometimes patients undergo chemotherapy near death, despite the studies that do not support this practice. For example, a recently published study showed that palliative chemotherapy was associated with worse quality of life in their last week of life and had no benefit to overall survival [20]. Newer drugs include molecular targeted agents (MTA), some of which are administered orally, may have survival benefits when treating patients with advanced solid tumors, but it is measured in months [21]. The criterion of tumor growth during anticancer treatment for discontinuing is complicated by reports of rebound progression on stopping therapy with MTA [21]. There are no precise guidelines that can help the withdrawal of anticancer therapy in clinical practice. However, decisions about anticancer treatment are beyond the scope of this article.

## 6. A proposal for Drug Deprescription

To follow a systematic method is a more efficient and safe way for deprescribing. Although some drugs may be removed simultaneously, it is preferable to do this step by step allowing possible symptoms caused by the cessation of a drug to be identified. Therefore, it would be useful to develop a strategy for deprescribing drugs in several steps.

**Step 0**

At this step there should be a reappraisal of the patient’s clinical situation including, performance status, prognosis, his/her understanding of the clinical situation, and setting treatment goals accordingly.

**First Step**

The first step would be to find out all the medications a patient is taking. A list of medications can be used, but lists omit current medication with a frequency that can be as high as >80% in older patients. The accuracy of the correct record of the medications that patients are taking improves significantly if patients are asked to bring all their medication to consultations [18].

**Second Step**

Deprescribing should be carried out with the agreement of the patient and carers. They should be informed of the purpose of the medications and the reasons for withdrawing some of them. Even if a patient is reluctant to stop taking a medication he/she has used for many years, in later consultations the patient may agree to discontinue it [18]. However, if the doctor deems a medication unsafe or unnecessary, he/she is not obliged to prescribe it.

**Third Step**

In this step, drugs which can be deprescribed in the first place without causing harm should be identified: drugs which are no longer being used or are duplicated; drugs which are causing relevant adverse effects; drugs prescribed for conditions that have been resolved; medication used for controlling side effects of another medication which has been discontinued, such as proton pump inhibitors prescribed for the prevention of gastroduodenal toxicity in patients taking nonsteroidal anti-inflammatory drugs which were discontinued [18].

**Forth Step**

In the fourth step, it is important to address medication that: requires a long time until benefit, outside of the patients’ expected lifespan such as statins that take more than one year to produce a benefit, being useless in patients with a short-expected survival time; has no proven benefit beyond a certain age, independent of the life expectancy of patients, such as bisphosphonates that do not protect women aged over 80 years and without previous hip or vertebral fractures from hip or wrist fractures [18].

**Fifth Step**

The fifth step is for the identification of medications that could be withdrawn, but slowly, because they would probably cause withdrawal or rebound symptoms or disease recrudescence with associated symptoms, that can become serious if discontinued abruptly, such as α-blockers, β-blockers, ACE-inhibitors, anti-anginal agents, anticonvulsants, antidepressants, anti-Parkinsonian agents, antipsychotics, anticholinergics, baclofen, benzodiazepines, corticosteroid, and digoxin [3]. Patients and families should be warned of the problems that can be anticipated and advised of what to do in case of occurrence of such a problem.

**Sixth Step**

The process of deprescribing should be monitored carefully in follow up appointments to identify clinical problems that may occur and to respond to misunderstandings and doubts about the process that may arise.

## 7. Conclusions

Inappropriate drug use in advanced cancer patients may cause harm and increases costs. It is important that health professionals recognize this problem, mainly doctors who have the responsibility for prescribing drugs, but other team members too, such as nurses and pharmacists who also have an important role. There are, however, several barriers to deprescription that may be not easy to overcome. The development of tools for identifying inappropriate medications and strategies for deprescription can help professionals dealing with those patients to reduce the irrelevant and the potentially harmful medication. To assist professionals to deal with this problem a six-step method is proposed.
